# Evaluation of a web-based portal to improve resident education by neonatology fellows

**DOI:** 10.3402/meo.v19.24403

**Published:** 2014-07-23

**Authors:** Ashwini Lakshmanan, Kristen T. Leeman, Dara Brodsky, Richard Parad

**Affiliations:** 1 Division of Newborn and Infant Critical Care, Children's Hospital Los Angeles, Keck School of Medicine, University of Southern California, Los Angeles, CA, USA; 2 Division of Newborn Medicine, Boston Children's Hospital, Harvard Medical School, Boston, MA, USA; 3 Harvard Stem Cell Institute, Boston Children's Hospital, Boston, MA, USA; 4 Division of Neonatology, Beth Israel Deaconess Medical Center, Harvard Medical School, Boston, MA, USA; 5 Division of Newborn Medicine, Brigham and Women's Hospital, Harvard Medical School, Boston, MA, USA

**Keywords:** WBEP, NICU, resident education, fellow-led teaching

## Abstract

**Background:**

Integration of web-based educational tools into medical training has been shown to increase accessibility of resources and optimize teaching. We developed a web-based educational portal (WBEP) to support teaching of pediatric residents about newborn medicine by neonatology fellows.

**Objectives:**

1) To compare residents’ attitudes about their fellow-led education in the NICU pre- and post-WBEP; including assessment of factors that impact their education and usefulness of teaching tools. 2) To compare fellow utilization of various teaching modalities pre- and post-WBEP.

**Design/methods:**

We queried residents about their attitudes regarding fellow-led education efforts and various teaching modalities in the NICU and logistics potentially impacting effectiveness. Based on these data, we introduced the WBEP – a repository of teaching tools (e.g., mock code cases, board review questions, journal articles, case-based discussion scenarios) for use by fellows to supplement didactic sessions in a faculty-based curriculum. We surveyed residents about the effectiveness of fellow teaching pre- and post-WBEP implementation and the type of fellow-led teaching modalities that were used.

**Results:**

After analysis of survey responses, we identified that residents cited fellow level of interest as the most important factor impacting their education. Post-implementation, residents described greater utilization of various teaching modalities by fellows, including an increase in use of mock codes (14% to 76%, *p*<0.0001) and journal articles (33% to 59%, *p=*0.02).

**Conclusions:**

A web-based resource that supplements traditional curricula led to greater utilization of various teaching modalities by fellows and may encourage fellow involvement in resident teaching.

Web-based tools are becoming critical supplements for training medical students, residents, and fellows. Benefits include the ability for standardization, flexibility for the student–teacher pair to select the time and place for learning, and ease of accessibility. These alternative teaching options are especially beneficial in the current environment of restricted resident work hours and decreased resident training time in Newborn Medicine per Accreditation Council for Graduate Medical Education (ACGME) guidelines. Studies have identified key subject areas best suited for Internet-based learning, including the critical care setting where learners can become familiar with rare events (1).

Although web-based tools for critical care learning are available, quality varies considerably. Prior to developing Internet-based learning tools, educators need to consider availability, relevancy, redundancy, and an approach for system modification (2). One study identified 135 web-based educational resources that utilized multiple modalities varying in utility and accuracy (2). A systematic review examined the effectiveness of web-based learning in health professionals’ education and found that interactivity, practice exercises, repetition, and feedback were associated with improved learning outcomes (3). In the absence of customized assessment tools, monitoring impact is challenging. Without proper guidance, the wide range of teaching options can be daunting for educators.

The goals of subspecialty fellowship training include development of skills as clinical leaders, researchers, and teachers. The teaching role of fellows requires mastery of subjects and problem-solving skills when challenged by engaged learners. However, fellows have limited formal teaching instruction and rarely receive feedback about their teaching skills. In addition, successful resident learning is dependent on both the residents’ and fellows’ level of interest. Several studies have cited that trainees benefit specifically from teaching by fellows (4). However, studies have also cited that fellows often lack a formalized curriculum or tools for teaching (5, 6). To help engage fellows in resident education, one program encouraged NICU fellows to develop and implement a new daily didactic curriculum (7). In this study, pediatric resident attitudes toward fellow teaching and the program after implementation were found to be more positive. Web-based modalities are an ideal adjunct to provide fellows with tools for teaching, foster fellow teaching skills, and enhance resident learning.

We created a web-based newborn medicine educational portal to enhance utilization of existing and newly created educational tools, and enhance the neonatology fellow teaching role. The specific aims of our project were 1) to compare residents’ attitudes about their fellow-led education in the NICU pre- and post-web-based educational portal (WBEP), including assessment of factors that impact their education and usefulness of teaching tools; 2) to compare fellow utilization of various teaching modalities pre- and post-WBEP.

## Methods

### Setting

We recruited pediatric residents from a large neonatal intensive care unit (NICU) at the Brigham and Women's Hospital (BWH) in Boston, Massachusetts. Residents and fellows rotate through this NICU from an affiliated tertiary care children's hospital (Boston Children's Hospital) from the Boston Children's Residency Program (BCRP) and the Harvard Neonatal–Perinatal Medicine Fellowship Program (HNPMFP).

The BCRP is ACGME accredited and comprised 150 pediatric residents, most of whom are required to complete 2 four-week NICU rotations (one at the BWH). During pediatric internship [i.e., post-graduate year one (PGY-1)], each PGY-1 rotated through this NICU as part of a team comprised of 1 neonatologist, 1 neonatology fellow, and 2–4 PGY-1s. This team provided clinical care to 16 Level 2 or 3 neonatal patients. Since 2011, the residency program has followed the ACGME 2011 Duty Hour Standard, limiting PGY-1 to a maximum of 16 continuous duty hours (8).

The ACGME-accredited HNPMFP trained 18 neonatology fellows per year. The first year of fellowship, included four one-month BWH NICU rotations including daytime shifts and overnight call. The second and third fellowship years consisted of 2–3 months of BWH night call coverage. During the fellow-led daily work rounds and at the bedside, neonatology fellows spent significant time interacting directly with residents and played a major role in resident education. Fellow responsibilities included direct patient care and oversight of the pediatric residents’ training. No formal scheduled teaching time or teaching tools had been delegated for fellow-led resident education prior to our intervention. The contribution of the fellow-resident teaching interactions had not been previously formalized within the curriculum.

### Baseline assessments

Given the coupling of decreasing exposure to clinical time in the NICU and an increasing role of the neonatology fellow as the supervising physician who spends the most time with the resident, resident education in the BWH NICU was becoming increasingly dependent on neonatology fellow-led teaching.

To assess both resident attitudes *and* utilization of teaching modalities, prior to implementation of our fellow-based teaching curriculum, we surveyed 49 residents who had rotated as PGY-1 residents during the 2009–2010 and 2010–2011 academic years. After characterizing resident teaching modality preferences during the 6 months prior to the intervention, a need was suggested for process improvement to develop a structured curriculum for fellow-led resident education.

### Study design

**Fig. 1:**
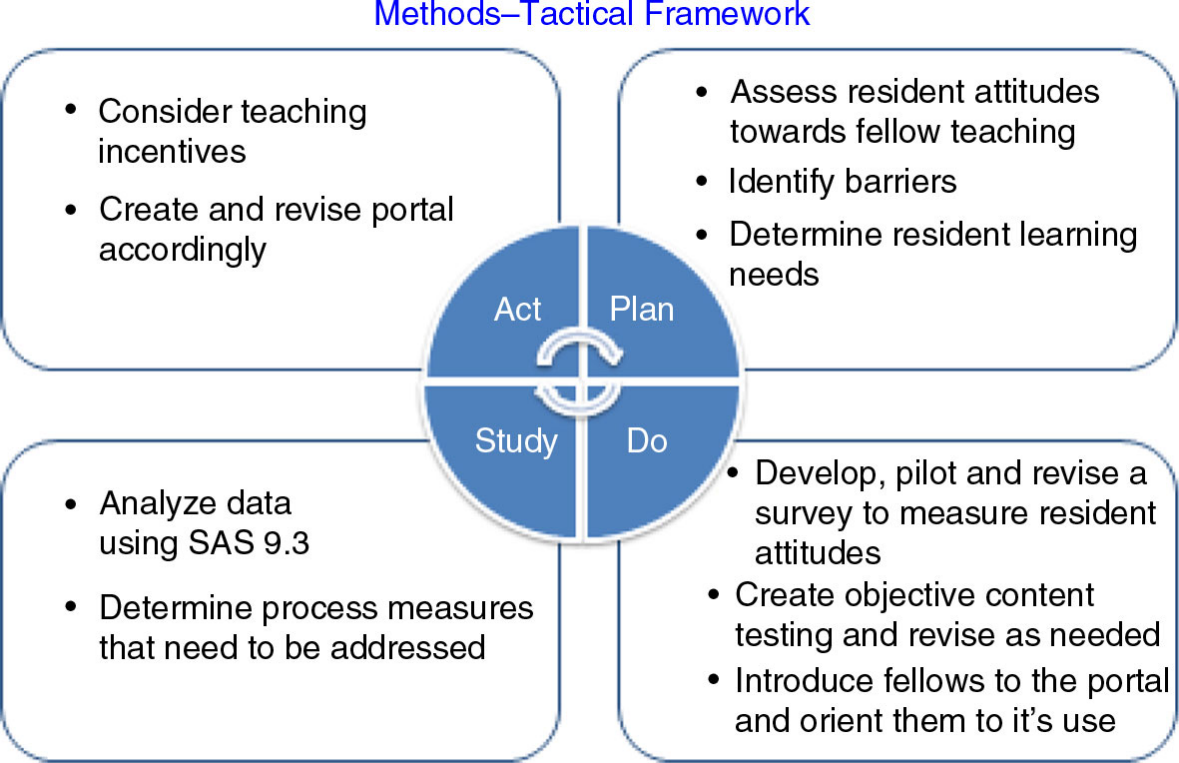
Tactical framework: Plan-Do-Study-Act (PDSA) cycle for intervention.

In the setting of the 2011 ACGME Duty Hour Standards altered duty hours for PGY-1 residents, we utilized a quality improvement Plan-Do-Study-Act (PDSA) tactical framework (Fig. 1) to improve resident education. In the planning component, we assessed resident attitudes about fellow teaching and identified barriers to teaching prior to the intervention. We realized that the residents’ educational program lacked both a formal structure for fellow-led resident education and also lacked easily accessible, comprehensive teaching tools.

We designed, piloted, and revised a cross-sectional quality improvement survey to evaluate resident attitudes about current education and teaching practices and then developed content and populated the WBEP with material guided by ACGME-recommended goals. We specifically addressed our first objective by querying residents about factors that both positively and negatively impact fellow-led education as well as usefulness of various teaching modalities. For our second objective, we also asked residents to identify the teaching modalities used by fellows. Because a validated assessment instrument does not exist, we created our survey by establishing face validity. In this approach, authors used a consensus-building approach to establish key questions that were relevant to NICU education. Subsequently, we determined process measures that addressed the utility of the WBEP. We developed the WBEP to provide educational prompts for fellows to utilize during impromptu resident teaching and scheduled weekly fellow-run mock codes. Usually, one fellow was paired with 2–3 residents for these teaching opportunities. The WBEP served as an easily accessible platform for the fellows to utilize different types of teaching modalities to educate the residents. For example, the portal provided sentinel neonatology articles with discussion questions, scripted simulation exercises, and case vignettes for the fellows to describe and discuss with the residents. The teacher and learner team could choose which teaching modality they preferred for each session. We promoted the WBEP in bi-weekly fellow meetings including introduction to the portal, review of the available resources, and encouragement of use for resident teaching.

### Intervention

The WBEP, a repository of resources, contained more than 110 resource pages including questions, case discussions, articles, mock codes, and knowledge tests (Table 1). The resources were designed for a teacher and learner team to review together with a focus on discussion. For the fellow-run mock code scenarios, scripted simulations were available on the WBEP. Scenarios included apnea in the well-baby nursery, acute hypovolemic shock, unplanned extubation in the NICU, and attendance of the delivery of a depressed newborn with meconium stained amniotic fluid. A regular, once weekly assigned time block was scheduled and advertised for implementing the mock codes, which the fellow could choose to utilize. We also included ‘virtual patient’ cases of classic NICU clinical vignettes with both question prompts for discussion and laboratory and radiographic findings, enhancing the interactivity of the educational content.

**Table 1:** Resources available on the web-based educational portal

Resource
American Academy of Pediatrics board preparation questions
Case-based discussion prompts
Infant follow-up program materials
Journal articles
Mock code simulation prompts
Neonatal imaging resources
Newborn nursery educational materials
Nutrition references
‘Virtual patient’ cases
Knowledge tests

We launched the WBEP in October 2011, 3 months after the 2011 ACGME Duty Hour Standards were implemented. The program was updated weekly and accessible to all BWH fellows and residents. The pre-implementation historical cohort was surveyed prior to the intervention in October 2011 and the post-implementation cohort was surveyed between October 2011 and June 2012.

### Participants

Our pre-implementation cohort included 49 pediatric residents who rotated in the BWH NICU during the 2009–10 or 2010–11 year as PGY-1 residents. The post-implementation cohort included 29 residents who were also PGY-1 residents who rotated in the BWH NICU after the implementation of the portal. All participants before and after the WBEP implementation were invited by e-mail to complete a cross-sectional quality improvement survey. Both samples were obtained by convenience sampling.

### Outcome measures

The primary outcomes assessed were resident attitudes about the impact of various factors on their neonatology education and usefulness of various teaching modalities in their education and the PGY-1 resident report of fellow teaching modality utilization.

We measured resident attitudes and teaching tool utilization using a 10-item cross-sectional quality improvement survey. The survey contained questions about participant characteristics, resident attitudes about factors impacting education, usefulness of teaching modalities, and rate of utilization of teaching modalities. After implementation of the WBEP, we surveyed the first 10 residents who had exposure to fellow-led use of the WBEP to complete our PDSA cycle 1. After examining resident feedback, we revised the WBEP by including additional PowerPoint presentations, journal articles, and clinical vignettes. We then recruited 19 additional residents who completed the cross-sectional quality improvement survey. We completed PDSA cycle 2 in June 2012. The same survey instrument was administered to the residents pre- and post-implementation to address both objective 1 and 2 of the study.

### Analysis

We conducted bivariate analyses to examine associations of predictors with outcomes using Chi-squared test for categorical and *t*-tests for continuous variables. We used SAS version 9.3 (SAS Institute Inc., Cary, NC) for analyses.

The study was deemed exempt from review by the Institutional Review Board because it was considered a quality improvement project.

## Results

A total of 78 residents responded to the quality improvement education survey (51% response rate) – 49 (54%) pre-implementation and 29 (64%) post-implementation. Resident participant characteristics are presented in Table 2. There was no significant difference between the pre- and post-intervention group in either the number of rotations as an intern, number of months since last rotation, training year of fellow, or attitude about effectiveness of fellow teaching.

**Table 2:** Resident and fellow participant characteristics

Resident characteristics	All participants (*n*=78) %	Residents before implementation (*n*=49) %	Residents after implementation (*n*=29) %	p#
Number of rotations in the NICU as an intern				
1	81	86	73	0.58
2	14	10	21	
Missing	3	4	0	
Number of months since last intern rotation				
0–6	46	25	83	0.46
7–12	15	21	7	
13–18	15	25	0	
>18	21	30	4	
Missing	3	0	6	
Year of training of fellow				
First	69	65	76	0.34
Second	6	4	10	
Third	5	8	0	
Hospitalist/Other	12	12	10	
Missing	8	11	4	
Resident attitudes toward effectiveness of teaching from neonatology fellows				
Not effective–slightly effective	11	6	21	0.12
Neutral	9	8	10	
Effective–very effective	80	86	69	

# p value significance<0.05; discerned by Chi-squared, Fisher's exact test.

To address our first objective of comparing residents’ attitudes about their fellow-led education in the NICU pre- and post-WBEP, we surveyed residents about factors that positively and negatively affected their education during the NICU rotation including: patient acuity, fellow responsibilities, fellow level of interest, resident schedule, resident level of interest, attending presence on rounds, and lack of access to teaching tools. We specifically addressed attitudes toward both ‘fellow level of interest’ and ‘resident level of interest’ to assess impact of both members of the teacher/learner pair, as both are critical components of a successful educational experience. Interestingly in the pre-implementation survey, as demonstrated in Table 3, fellow level of interest was cited as the factor that *most* positively (60% of all respondents) and *most* negatively (60% of all respondents) affected their NICU education. In addition, resident reflections showed that resident level of interest positively impacted their education (56%), while fellow responsibilities (51%) and resident schedules (46%) had a negative effect. The majority of residents (80%) rated fellow teaching as effective. Post-implementation, similar results were obtained as pre-implementation survey results, including a continued highlight on fellow level of interest as a critical component (see Table 3). Comparing attitudes pre- and post-intervention, the negative impact of lack of access to teaching tools was more evident in the pre-implementation compared with the post-implementation group (25% vs. 7%, *p=*0.06).

**Table 3:** Resident attitudes toward various factors negatively and positively impacting their education

	All participants (*n*=78)	Residents before implementation (*n*=49)	Residents after implementation (*n*=29)	
Factor	%	%	%	p#
Patient acuity				
Negatively impacts	28	35	17	0.11
Positively impacts	33	27	45	0.15
Fellow responsibilities				
Negatively impacts	51	57	41	0.21
Positively impacts	6	8	3	0.41
Fellow level of interest				
Negatively impacts	60	61	58	0.9
Positively impacts	60	59	62	0.82
Resident schedule				
Negatively impacts	46	41	55	0.33
Positively impacts	12	10	14	0.64
Resident level of interest				
Negatively impacts	15	16	14	0.66
Positively impacts	56	53	62	0.44
Attending presence on rounds				
Negatively impacts	9	8	10	0.83
Positively impacts	17	20	10	0.25
Lack of access to teaching tools				
Negatively impacts	17	25	7	0.06

# p value significance<0.05; discerned by Chi-Squared, Fisher's exact test.

As a second component of our first objective to assess resident attitudes toward fellow-led education, we asked residents’ about the usefulness of various teaching modalities. After PDSA cycle 1, we found that residents (*n*=10) surveyed post-WBEP implementation identified the following modalities as useful: mock codes (100%), journal articles (70%), case-based discussions (100%), and bedside teaching (100%) (Table 4). Given these findings, we increased the number of teaching tools available for mock code/simulations, journal article discussions, and clinical case presentations on the WBEP. Additionally, we updated the user interface to improve our presentation of the case vignettes and continued encouragement of fellow use of the WBEP via reminders at biweekly fellow meetings. Subsequently, we completed PDSA cycle 2 (*n*=19) in our outcome analysis. Combining our results from PDSA cycle 1 and 2, we found that residents rated usefulness of journal articles more highly after the WBEP implementation (59% vs. 83%, *p=*0.01).

**Table 4:** Resident report of usefulness of various teaching modalities

Report of usefulness of each teaching modality	All participants (*n*=78)%	Residents before implementation (*n*=49)%	Residents after implementation (*n*=29)%	p#
Mock Codes				
Not useful–slightly useful	4	4	4	0.81
Neutral	5	7	4	
Useful–very useful	82	78	89	
Missing	9	11	3	
Board review questions				
Not useful–slightly useful	16	16	14	0.91
Neutral	28	27	31	
Useful–very useful	42	41	45	
Missing	14	16	10	
Journal articles				
Not useful–slightly useful	5	4	7	0.01
Neutral	15	24	0	
Useful–Very useful	68	59	83	
Missing	12	13	10	
Case based discussions				
Not useful–slightly useful	3	2	3	0.51
Neutral	3	4	0	
Useful–Very useful	84	84	87	
Missing	10	10	10	
Bedside teaching				
Not useful–slightly useful	1	0	4	0.19
Neutral	0	0	0	
Useful–very useful	87	88	86	
Missing	12	12	10	

# p value significance<0.05; discerned by Chi-squared, Fisher's exact test.

To measure our second objective of comparing fellow utilization of various teaching modalities, we queried residents about the utilization of various teaching modalities both pre- and post-implementation. Interestingly, although the residents cited that each tool was useful, they also reported that they were not being utilized frequently (range between 12 and 33%) prior to WBEP implementation (Table 5). Using our pooled results from PDSA cycle 1 and 2 (pre-intervention *n=*49, post-intervention *n*=29), we found a significantly greater fellow-led utilization of several queried teaching modalities (Table 5). Specifically, there was a reported increase in use of mock codes (14–76%, *p*<0.0001) and journal articles (33–59%, *p=*0.02) by fellows.

**Table 5:** Resident report of utilization of various teaching modalities

Utilization of each teaching modality	All participants (*n*=78)	Residents before implementation (*n*=49)	Residents after implementation (*n*=29)	
	%	%	%	p#
Mock codes	37	14	76	<0.0001
Board review questions	9	12	3	0.19
Journal articles	42	33	59	0.02
Case based discussions	22	24	17	0.48
Bedside teaching	68	71	62	0.47

# p value significance<0.05; discerned by Chi-squared, Fisher's exact test.

## Discussion

We describe a successful educational quality improvement intervention, which created a structured teaching role for fellows to supplement an existing curriculum that led to increased utilization of various teaching modalities. We propose that the success of the intervention was the result of the following factors:

1. Accessibility of the WBEP. The WBEP was available online from any computer in the NICU to all fellows and residents.
2. Straightforward user interface. The WBEP user interface was very easy to navigate for the fellow as teacher. The toolbars were highlighted in a different color making it simple to find various resources.
3. Interactivity. Addition of ‘virtual patient’ cases provided a platform for interactive PGY-1 patient assessment and management with the fellow taking the role of facilitator.
4. Accuracy and quality. The WBEP included neonatology focused high quality resources and tools. The computer-based program permitted weekly modifications, allowing incorporation of the most up-to-date evidence-based materials.
5. Encouragement and recognition of the fellow teaching role. WBEP development and focus on the NICU fellow teaching role led to an increased emphasis on fellow teaching. This increased awareness and recognition alone likely contributed to the success of fellow-led resident teaching.

Our first objective, comparing resident's attitudes toward fellow-led education pre- and post-intervention, identified fellow level of interest as a key factor affecting the resident NICU educational experience. While fellow level of interest in teaching is not an easily modifiable factor, we hypothesized that ease of WBEP accessibility, variety of available teaching tools, scheduled weekly teaching session and ongoing promotion of the WBEP may help foster fellow interest in education and lead to better resident education. The residents’ attitudes toward effective teaching tools likely depended on each fellow's modality preference for teaching. While this cannot be accounted for in our analysis, the generalizability remains robust as the WBEP is designed for a wide-range of fellow/teacher resident/learner pairs, both likely to have varied preferences. Our goal to provide a variety of reliable, accessible teaching tools where the teacher/learner pair can pick their modality appears achievable for application in clinical academic medical education.

Our second objective compared the utilization of different teaching modalities by the fellows pre- and post-intervention and found an increased use of mock codes and journal articles post-intervention. The increased mock-code utilization may have been stimulated by providing a scheduled weekly blocked time for a potential mock-code session. The increased use of journal articles for teaching occurred at the discretion of the fellow/resident pair. We can hypothesize that the increased use of journal articles may have been due to ease of access to sentinel and state-of-the-art neonatology articles on the WBEP.

One limitation of this intervention is that this is a single site study. However, the material provided in the WBEP could be generalizable and made accessible to other academic institutions to supplement their neonatology fellow teaching tools for resident education. Another limitation was that the survey measure was not piloted or administered to a focus group. However, it did have face validity as it was reviewed multiple times by the authors. The WBEP was specifically designed to focus on fellow-led teaching tools to enhance interactive teaching, rather than replacing faculty teaching or resident self-learning. While this set-up was by design, it could be seen as a limitation because of the missed opportunity for resident self-learning. However, the WBEP could be easily amended to include an additional self-learning focused section to address this issue.

Each fellow/resident pair had different levels of exposure to the WBEP. The fellow teaching curriculum implemented with the WBEP included only one scheduled weekly session. The remainder of the fellow teaching time was dependent upon fellow and resident level of interest, acuity of patient responsibilities and time constraints. While a strict curriculum was not enforced, we believe that our intervention is generalizable to a realistic NICU teaching environment with opportunities for impromptu teaching sessions arising at different periods of time throughout a resident's rotation.

Only a few publications address quality improvement initiatives in pediatric residency education (9–11). Baron et al. examined the impact of fellow teaching on resident education after implementation of a locally designed web-based resource (9). Our results similarly suggest that using online resources may have a significant role in supplementing traditional curricula during an era of restricted duty hours.

We demonstrated that a web-based resource that supplemented traditional curricula encouraged fellow-led utilization of various teaching modalities. Utilization of web-based teaching tools presents the opportunity for continued improvement in both fellow teaching skills and resident education. In our current age of technology, teaching using web-based resources will likely become a critical component of medical training. Ongoing work is needed to develop accurate, accessible resources to test the quality of these resources and to assess their impact on resident learning.
